# WE CANNOT WALK THERE ANYMORE: EXPERIENCES OF URBAN STRESS IN AGE-FRIENDLY ENVIRONMENTS

**DOI:** 10.1093/geroni/igae098.0758

**Published:** 2024-12-31

**Authors:** Ad Maulod, Malcolm Ravindran, Yunjie Wong

**Affiliations:** Duke-NUS Medical School, Singapore, Singapore; Duke-NUS Medical School, Singapore, Singapore; Duke-NUS Medical School, Singapore, Singapore

## Abstract

Urban stress, or tension attributed to city living, is often experienced by individuals in urban areas due to environmental and social stressors. The life-space framework assesses older persons’ mobility based on the extent of movement within their environment while engaging in activities, and whether assistance is required for said movement. Chronic exposure to urban stress affects older persons’ life-space, and is associated with reduced quality of life. Yet, the connection between urban stress and life-space has not been fully explored in the existing literature. Singapore is the third most densely populated city in the world, ranking highly in terms of liveability. However, the country also undergoes frequent redevelopment, which presents a source of urban stress for older Singaporeans who must continually adapt to changing and unfamiliar landscapes. This presentation draws from ‘go-along’ and in-depth interviews with 60 older Singaporeans. Findings highlight challenges in the lived environment and the difficulties individuals experience travelling to places and activities that are important to their well-being. Despite the emphasis on developing age-friendly infrastructure in Singapore, incompatible person-environment fit was evident among participants who struggle with accessibility, wayfinding, social exclusion, heat and poor home ventilation, care options, and lack of choices in living situations. These issues limit older persons’ life-space capacities and hinders aspirations to age well. Policies promoting ageing-in-place must (i) consider multi-level, intersectional factors comprising built environments, individual resources and social contexts that may mitigate frailty and urban stress, and (ii) involve older persons in designing environments which promote autonomy and belonging.

